# Bevacizumab Treatment for Meningiomas in NF2: A Retrospective Analysis of 15 Patients

**DOI:** 10.1371/journal.pone.0059941

**Published:** 2013-03-21

**Authors:** Fabio P. Nunes, Vanessa L. Merker, Dominique Jennings, Paul A. Caruso, Emmanuelle di Tomaso, Alona Muzikansky, Fred G. Barker, Anat Stemmer-Rachamimov, Scott R. Plotkin

**Affiliations:** 1 Neurology Department, Massachusetts General Hospital, Boston, Massachusetts, United States of America; 2 Genetics Department, Massachusetts General Hospital, Boston, Massachusetts, United States of America; 3 Stephen E. and Catherine Pappas Center for Neurooncology, Massachusetts General Hospital, Boston, Massachusetts, United States of America; 4 A.A. Martinos Center for Biomedical Imaging, Massachusetts General Hospital, Boston, Massachusetts, United States of America; 5 Department of Radiology, Massachusetts General Hospital, Boston, Massachusetts, United States of America; 6 Pathology Department, Massachusetts General Hospital, Boston, Massachusetts, United States of America; 7 Biostatistics Center, Massachusetts General Hospital, Boston, Massachusetts, United States of America; 8 Neurosurgical Service, Massachusetts General Hospital, Boston, Massachusetts, United States of America; University of California-San Francisco, United States of America

## Abstract

Bevacizumab treatment can result in tumor shrinkage of progressive vestibular schwannomas in some neurofibromatosis 2 (NF2) patients but its effect on meningiomas has not been defined.

To determine the clinical activity of bevacizumab against NF2-related meningiomas, we measured changes in volume of meningiomas in NF2 patients who received bevacizumab for treatment of progressive vestibular schwannomas. A radiographic response was defined as a 20% decrease in tumor size by volumetric MRI analysis. In addition, we determined the expression pattern of growth factors associated with tumor angiogenesis in paraffin-embedded tissues from 26 unrelated meningiomas. A total of 48 meningiomas in 15 NF2 patients were included in this study with a median follow up time of 18 months. A volumetric radiographic response was seen in 29% of the meningiomas (14/48). Tumor shrinkage was not durable: the median duration of response was 3.7 months and the median time to progression was 15 months. There was no significant correlation between pre-treatment growth rate and meningioma response in regression models. Tissue analysis showed no correlation between tumor microvascular density and expression of VEGF pathway components. This data suggests that, in contrast to schwannomas, activation of VEGF pathway is not the primary driver of angiogenesis in meningiomas. Our results suggest that a minority of NF2-associated meningiomas shrink during bevacizumab therapy and that these responses were of short duration. These results are comparable to previous studies of bevacizumab in sporadic meningiomas.

## Introduction

Meningiomas are the most common type of brain tumor, accounting for 34% of all central nervous system tumors.[1] Despite the high prevalence of meningiomas in the general population, there are currently no medical treatments available.[2], [3] For sporadic meningiomas that require active treatment, surgery and radiation therapy are usually effective. Meningiomas are even more common in neurofibromatosis 2 (NF2) patients, with a cumulative incidence of 80% by age 70,[4] and are a major cause of morbidity and mortality in these patients.[5], [6] The lack of effective medical therapy for meningiomas represents a significant challenge in the clinical management of NF2 patients. Unlike patients with sporadic tumors, NF2 patients often have multiple meningiomas, vestibular schwannomas, and ependymomas. The multiplicity of tumors make surgery and radiation therapy for all tumors impracticable.

Neovascularization is necessary for tumor growth beyond 2 – 3 mm,[3] the point at which diffusion alone becomes insufficient to meet basic tumor metabolic requirements,[7] and is driven by tumor produced angiogenic factors such as vascular endothelial growth factor (VEGF) that stimulate the growth of tumor capillaries. Bevacizumab is a humanized monoclonal antibody that neutralizes the activity of VEGF.[8] Bevacizumab prevents the binding of all VEGF isoforms to VEGF receptors, and is currently approved by the Food and Drug Administration (FDA) for clinical use in recurrent glioblastoma, metastatic colorectal cancer, advanced nonsquamous non-small cell lung cancer, and metastatic kidney cancer (www.fda.gov on 02/05/2012).

We have recently shown that treatment with bevacizumab can lead to hearing improvement and tumor shrinkage in some NF2 patients with progressive vestibular schwannomas.[9] Tissue analysis of schwannomas suggested activation of the VEGF pathway due to decreased expression of SEMA3, an angiogenesis inhibitor. The effects of bevacizumab on meningiomas are not clear. To date, two case reports and two case series have been published on bevacizumab use in intracranial meningiomas, with anecdotal reports of meningioma response to bevacizumab.[10]–[13] We present here a retrospective analysis of tumor response in 48 intracranial meningiomas from 15 NF2 patients treated with bevacizumab for progressive vestibular schwannoma.

## Methods

### Ethics Statement

This research study was approved by the Partners Human Research Committee Institutional Review Board. Requirement for informed consent was waived for this retrospective analysis of clinical data.

### Patients

Between 2007 and 2011, a total of 31 NF2 patients were treated at our center using bevacizumab for progressive vestibular schwannomas. Of these 31 patients, 16 also had intracranial meningiomas (55%). Two patients were excluded from the study because of incompatibility between the MRI scan format performed at an outside facility and our volumetric analysis software. We included one additional NF2 patient who underwent surgical resection of bilateral vestibular schwannomas and was treated using bevacizumab for a single progressive meningioma. A total of 48 meningiomas and 18 vestibular schwannomas in 15 NF2 patients were included in the analysis. Patients received bevacizumab 5 mg/kg I.V. every 2 weeks as part of clinical care for their vestibular schwannoma. Higher doses used for malignant brain tumors (e.g., 10 mg/kg) were not used in order to minimize risk of toxicity. Brain MRI scans were performed within about 1 month prior to start of treatment, and approximately every 3 months after the start of treatment to monitor tumor response. Adverse events were classified according to the Common Terminology Criteria for Adverse Events, version 4.

### Volumetric analysis

Commercial software (Alice; Hayden Image Processing Group/Parexel International Corp, Waltham, MA) was used to create the volumetric measurements on contrast enhanced T1-weighted axial images. Once outlining was complete, actual volume measurements were calculated in Matlab (The MathWorks, Natick, MA) by counting the number of voxels in a given image and multiplying the count by the volume of a single voxel (as calculated using the in-plane and through-plane image resolution). A maximum number of eight tumors per patient can be measured using the Alice software. In patients with more than 8 tumors, volumetric analysis was first performed on vestibular schwannomas and then on meningiomas (from largest to smallest). Total intracranial tumor volume was defined as the sum of the volume of all intracranial meningiomas measured in a patient. We used clinically indicated MRIs performed prior to start of bevacizumab therapy to determine pre-treatment meningioma annual growth rate (AGR). Tumor growth rate was defined as (tumor volume – baseline tumor volume)/ baseline tumor volume.

Tumor contours for volumetric analysis were delineated by a single researcher (FN) who was blinded for patient identity, time of scan, and treatment status (pre- or post-bevacizumab). All tumor contours were performed using T1 post-contrast axial MRI scans. After contours were completed, a neuroradiologist (PC) with 10 years of post-fellowship academic experience reviewed the contours. For tumors with indistinct boundaries, the neuroradiologist reviewed axial, sagittal, and coronal slices of pre- and post-contrast T1-weighted, T2-weighted, diffusion-weighted images, FLAIR, susceptibility, FIESTA images, and CT scans when available. Details of the methodology used for determining tumor contours are available in the supplemental material (Text S1).

### Definition of imaging response

A radiographic response was defined as a decrease in tumor volume of ≥20% at any time compared with baseline volume. Tumor progression was defined as an increase in volume of ≥ 20% when compared to baseline. Stable disease included all other responses. A sustained radiographic response was defined as a decrease in tumor volume of ≥ 20% when compared to baseline at the time of the last scan. Duration of response was determined as the time interval from the first and last scans showing a decrease in volume ≥ 20% from baseline, even if interim scans did not meet radiographic response criteria. Time to progression was defined as the time from first dose of bevacizumab to the first scan date in which the meningioma grew ≥ 20% in volume compared to baseline measurements.

### Regression Analysis

We analyzed each outcome of interest on a per-tumor basis and on a per-patient basis. In the per-tumor analysis, each tumor was considered an independent event with no clustering effect for individual patients with multiple tumors. In the per-patient analysis, we accounted for the clustering effect of multiple tumors in individual patients by using a regression model that included a random effect. Details on the regression model used are available in the supplemental material. In both analyses, we calculated the tumor growth rate during the pre- and post-treatment periods using all scans available (up to 4 years before initiation of treatment). We also performed the analyses using only the scans in the 12 months immediately prior to initiation of therapy to determine pre-treatment annual tumor growth rate. We wanted to determine whether relative change in tumor volume (percent change from baseline) or absolute change in tumor volume would correlate with radiographic response to treatment. Therefore, tumor growth rate was represented as relative change in tumor volume from baseline (percentage response) and as absolute difference in tumor volume from baseline. We then correlated pre-treatment growth rates with post-treatment growth rates using patient demographic information (age at the start of treatment and gender), tumor size at baseline, and tumor location as covariates. Statistical analysis was performed with SAS software (version 9.2, SAS Institute Inc, NC, USA). A P value of <.05 was considered to be statistically significant.

### Immunohistochemistry

In a parallel analysis, we determined the expression pattern of growth factors associated with tumor angiogenesis in paraffin-embedded tissues of 13 unrelated NF2-related meningiomas and 13 sporadic meningiomas from surgeries at Massachusetts General Hospital. A similar analysis for NF2-related and sporadic schwannomas was reported previously.[9]Five micron-thick sections were cut and immunostained with the following antibodies: CD31 (Dako, prediluted), αSMA (Sigma, 1∶100), VEGF (Santa Cruz or Neomarker 1∶100), VEGFR2, PDGFR-α, PDGFR-β (Cell Signaling; 1∶250, 1∶100 and 1∶100 respectively), Neuropilin-1 (Chemicon, 1∶40), Neuropilin-2 (R&D Biosystem, 1∶500), Semaphorin 3A (Millipore, 1∶100) and Semaphorin 3F (Chemicon 1∶250). Semiquantitative analysis was performed by two authors who scored the intensity of staining of tumor cells and blood vessels on a scale from 0 (no staining) to 3 (strong staining). For calculation of microvascular density and diameter, CD31 labeling was used to highlight vessels. The quantification used at least 5 fields of confirmed tumor tissue at 200x magnification with an average of 100 vessels counted per section. A customized software analysis tool compatible with Image J (http://rsb.info.nih.gov/ij/) was then used to determine the number of vessels, perimeter, the minor axis of best-fitted ellipse (representative of the vessel diameter), and the total surface covered by vascular spaces. The same method was used substituting either VEGFR2 or NRP2 labeling to determine the percentage of vessels expressing these VEGF receptors. Consecutive sections were used to allow similar areas of the tumor to be quantified in all cases.

## Results

A total of 48 meningiomas and 18 vestibular schwannomas from 15 NF2 patients (7 men, 8 women) were included in this study. Patient and tumor characteristics are listed in Table 1. The mean number of MRI scans per patient used for analysis of tumor response was 8 (range, 4 – 14). The median patient age at the start of treatment was 29.5 years (range, 16 – 63 years). The mean (median) volumes of meningiomas and vestibular schwannomas at the beginning of treatment were 6.2 cm^3^ (1.9 cm^3^), and 9.8 cm^3^ (4.3 cm^3^), respectively. The mean volumetric growth rate prior to treatment was 11.6% per year and median follow up time was 18 months (range 10 to 37 months). Bevacizumab was overall well tolerated, with only four grade 3 adverse events (hypertension, elevated liver enzymes, menorrhagia, irregular menses), and two grade 4 events (both associated with wound healing problems, including vascular access complications and tracheostomy wound healing delays).

**Table 1:** Patient and tumor characteristics.

Characteristics	N (%)
Gender	
Female	8 (53%)
Male	7 (47%)
Age	
< 20y	2 (13%)
20 – 29y	7 (47%)
30 – 39y	2 (13%)
40 – 49y	3 (20%)
>50y	1 (7%)
Total meningiomas analyzed per patient	
1 – 2 meningiomas	8 (53%)
3 – 4 meningiomas	3 (20%)
>4 meningiomas	4 (27%)
Median meningioma baseline volume	1.9 cm^3^
Distribution of meningioma volume at baseline (n = 48)	
< 2 cm^3^	25 (52%)
2 – 10 cm^3^	16 (34%)
10 – 20 cm^3^	4 (8%)
> 20 cm^3^	3 (6%)
Meningioma Pre-Treatment Annual Growth Rate	11.6% (range,-240% to 546%)
Meningioma location	
Orbital/Skull base	10 (21%)
Convexity/Falx	34 (71%)
Posterior fossa	4 (8%)
Median vestibular schwannoma volume at baseline	4.3 cm^3^
Distribution of vestibular schwannoma volume at baseline (n = 18)	
< 2 cm^3^	5 (27%)
2 – 10 cm^3^	7 (39%)
10 – 20 cm^3^	3 (17%)
> 20 cm^3^	3 (17%)

**Figure 1:**
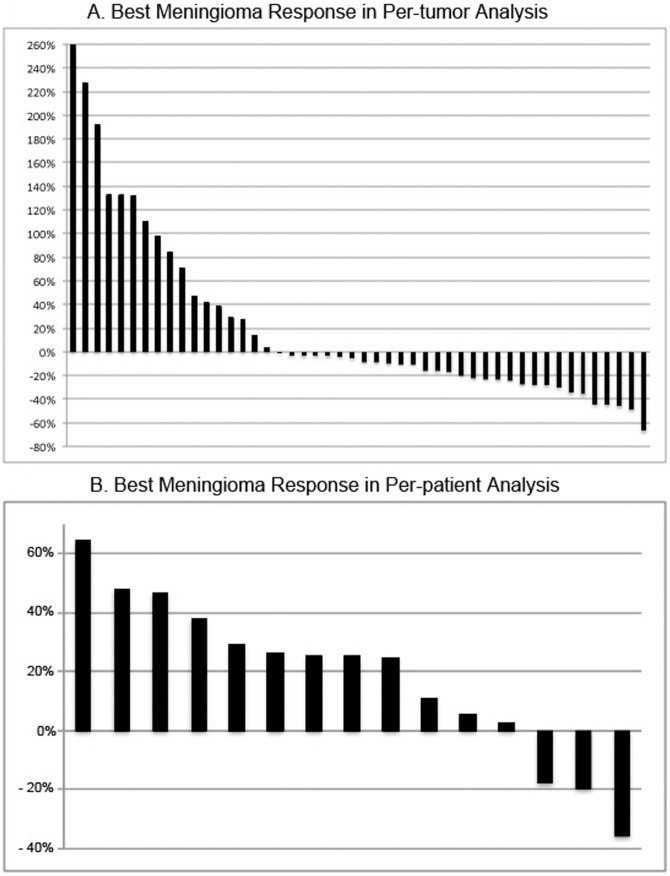
Radiographic Response of Meningiomas to Bevacizumab. Waterfall plot of best radiographic response for individual meningiomas (A) and for total meningioma volume in patients (B).

**Figure 2:**
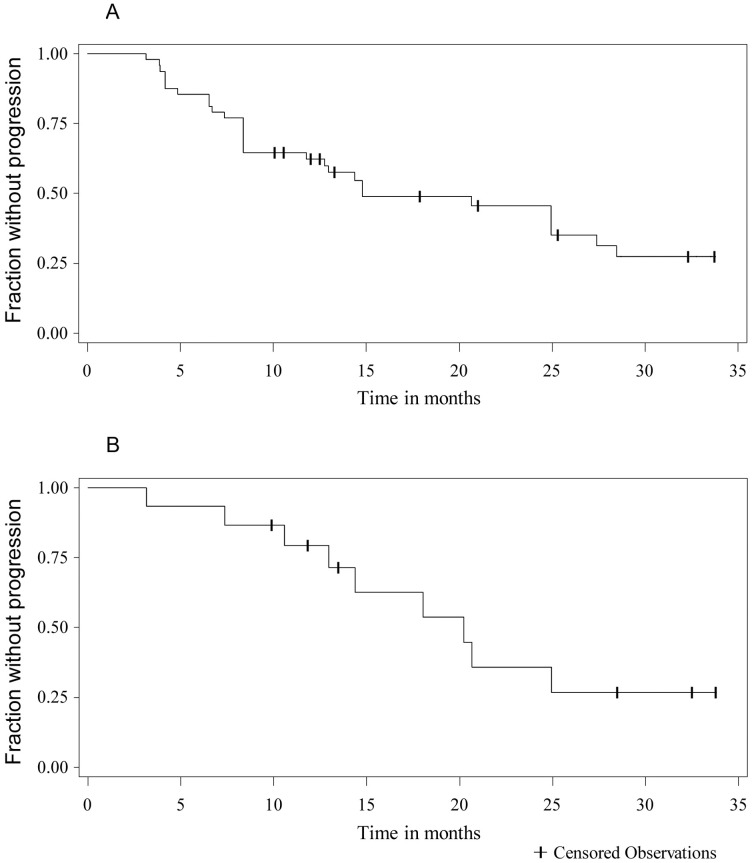
Time to Progression for Meningioma Treated with Bevacizumab. A. Kaplan-Meier curve for time to progression considering each meningioma as an independent event (per-tumor analysis). Progression was considered as an increase in tumor volume of 20% or more. B. Kaplan-Meier curve for time to progression using total intracranial meningioma volume for each patient as an independent event (per-patient analysis). Time to progression was 15 months for individual tumors and 20 months on per-patient analysis. Progression-free rate at six months was higher in the per-patient analysis at 93%, compared to 85% for the per-tumor analysis.

A radiographic response was seen in 29% of tumors (14/48 tumors) using per-tumor analysis and was seen in 7% of patients (1/15 patients using a per-patient analysis) (Figure 1). Tumor shrinkage was not durable, with only 5/14 responding meningiomas maintaining a radiographic response at the last follow up. The median duration of meningioma response was 3.7 months (range 0 to 25 months), with six meningiomas having a single scan meeting criteria for radiographic response (decrease in meningioma volume ≥ 20%). Overall, 29/48 meningiomas (60%) progressed during the study. The median time to progression was 15 months on per-tumor analysis (Figure 2) and was 20 months on per-patient analysis. Progression–free survival at 6- and 12-months was 85% and 62%, respectively, on per-tumor basis. Progression-free survival at 6- and 12-months was 93% and 79%, respectively on per-patient basis.

The radiographic response rate for vestibular schwannomas in this cohort was 39% (7/18 tumors) when using whole brain MRI scans and was 44% (8/18 tumors) when using internal auditory canal (3mm slices, no gap). The difference in response rate between the cohort of NF2 patients with meningiomas (44% response) and the entire treated NF2 cohort of 31 patients (55%, data not shown) was not statistically significant (Fisher exact two tail  =  .79).

**Figure 3:**
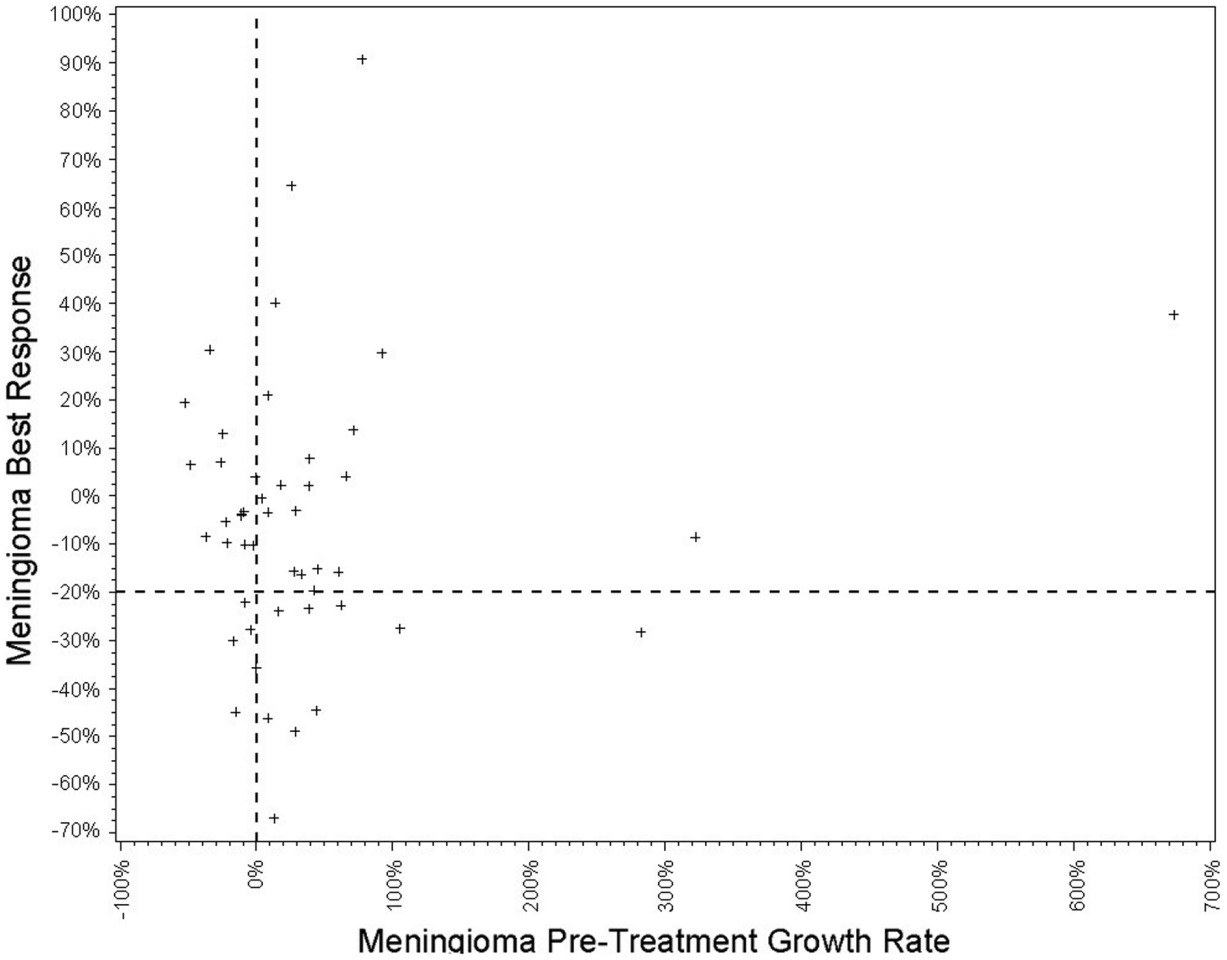
Meningioma Best Response Compared to Pre-treatment Growth Rate. Scatter-plot of meningioma best response vs. relative change in tumor volume prior to treatment. Horizontal dashed lines show threshold for radiographic response at 20% decrease in tumor volume post treatment, and vertical dashed line separates tumors with positive growth prior to therapy.

A total of 47 meningiomas were included in regression analyses to identify clinical factors associated with post-treatment growth rate. For one meningioma, no pre-treatment scans were available for analysis. Growth rate estimates and statistical results are summarized on Table 2. There was no significant correlation between pre-treatment and post-treatment growth rates for individual meningiomas using per-tumor analysis (*p*  =  .33). This finding persisted when pre-treatment growth was calculated using only the 12 months immediately prior to treatment (*p*  =  .79). There was no significant difference in the results when tumor response was calculated using absolute change in tumor volume or percent change in tumor volume (*p*  =  0.72). When only meningiomas with a pre-treamtent growth rate ≥ 20% and with baseline volume ≥ 1 cm^3^ were included in the analysis, radiographic response was seen in only one out of five meningiomas (20% of tumors). Figure 3 shows the correlation between meningioma best response and pre-treatment growth rate.

**Table 2:** Summary of Regression Analysis Results for Growth Rate Estimates in Per-Tumor and Per-Patient Models.

	Per-tumor Analysis				Per-patient Analysis			
Factor	Percentage Change in Tumor Volume		Absolute Change in Tumor Volume		Percentage Change in Tumor Volume		Absolute Change in Tumor Volume	
	*Parameter Estimate*	*p-value*	*Parameter Estimate*	*p-value*	*Parameter Estimate*	*p-value*	*Parameter Estimate*	*p-value*
Tumor Growth by Month Using All Scans	−0.053	0.33	−0.011	0.72	−0.066	0.23	−0.025	0.37
Tumor Growth by Month Using last 12 months	−0.014	0.79	0.002	0.94	−0.016	0.56	−0.04	0.43
Male Gender	N/A	N/A	N/A	N/A	−0.015	0.07	−0.06	0.33
Age	N/A	N/A	N/A	N/A	−0.0007	0.04	−0.004	0.09

In the per-patient analyses, there was no significant association between pre-treatment tumor growth and tumor response when using percentage change in tumor volume (*p  = * .23), or absolute change in tumor volume (*p*  =  .37). There was no difference in the results when pre-treatment period included only the 12 months immediately prior to start of therapy. Interestingly, there was a correlation between increased post-treatment growth rate and male gender (*p*  =  .07), and older age (*p*  =  .04).

Immunohistochemical analysis of meningiomas showed no significant difference in the expression of components of the VEGF angiogenesis pathway (VEGF, VEGFR2, PDGFR-α, PDGFR-β, neuropilin-1, neuropilin-2, semaphorin 3A, and semaphorin 3F) between sporadic meningiomas and NF2-associated meningiomas. Microvascular density was significantly greater in sporadic meningiomas compared to NF2-related meningiomas (52 vessels/mm2 vs. 32 vessels/mm2; t-test *p*  =  .01). There was no significant difference of average perimeter or diameter between the two groups.

## Discussion

Medical treatments for meningiomas are desperately needed for patients with meningiomas that cannot be adequately treated with surgery and radiation. The list of drugs without a clear benefit for these tumors continues to grow and includes tamoxifen,[14] irinotecan,[15] temozolomide,[16] octreotide,[17], [18] mifepristone,[19] erlotinib,[20] gefitinib,[20] imatinib,[21] and alpha-interferon.[22], [23] Although initial results with hydroxyurea were promising,[24] further studies showed that radiographic response to hydroxyurea in meningiomas is uncommon.[25], [26]

Our results for NF2-related meningiomas are comparable to case series of sporadic meningiomas treated with bevacizumab [12], [13]. These studies, which included all grades of meningiomas, reported median PFS of 18−26 months and radiographic response rates of 0−7%. Because multiple meningiomas are common in NF2 patients, we analyzed our data on a per-tumor basis and on a per-patient basis. Using per-tumor analysis, the median PFS was 15 months; using per-patient analysis, the PFS was 20 months. The per-tumor radiographic response rate was 29% in our series and exceeded the rate in previous reports; however, the durability of response was short. This contrasts with the durability of response in schwannoma.[9] These results suggest that the clinical benefit of tumor shrinkage in our patients was minimal.

Analysis of tumor growth rate can be determined as a relative change (percentage) in tumor volume from baseline, or as an absolute volume change from baseline measurement. The use of relative change is the standard method used for radiographic response to treatment, whether linear measurements or volumetric analysis methods are used in the study.[27] We analyzed our data in both ways to account for potential bias in the determination of response rate based on tumor size at baseline. Relative changes in tumor volume are usually larger in small tumors, leading to bias in the statistical analysis when smaller and likely asymptomatic tumors are included in the study. Instead, larger tumors are more likely to have larger absolute volume changes than small tumors, even if only a small percentage change in total volume has occurred. Due to the biology of VEGF inhibitors, such as bevacizumab, we decided to determined response rate using both relative change in tumor volume, as well as absolute changes in volume from baseline for comparison. In this retrospective analysis, neither measurement of pre-treatment growth rate was found to be significantly associated with treatment response. This may be due to poor response to treatment seen in this study, small sample size, or selection bias from this retrospective study. Although there was a statistical association between older patients and meningioma response to treatment, the relative effect on response was small, and likely not clinically relevant.

We have previously shown that treatment with bevacizumab can lead to tumor shrinkage of vestibular schwannomas in patients with NF2,[9] with follow up study showing radiographic response in 55% of vestibular schwannomas, and hearing improvement in 57% in our cohort of 31 patients.[28] In these same patients, radiographic responses for meningiomas were seen in only 29% of meningiomas and were short-lived.. The difference in response rate between vestibular schwannomas and meningiomas does not seem to correlate with degree of angiogenesis, since expression of VEGF in the tumor, expression of VEGFR2 in tumor blood vessels, and microvascular density are greater in meningiomas than in vestibular schwannomas.[9] However, immunohistochemical analysis of expression of the VEGF pathway components shows different patterns in schwannomas and meningiomas. In our previous study, analysis of the VEGF angiogenesis pathway in schwannomas showed very low expression of SEMA3 (angiogenesis inhibitor) and a positive correlation between VEGF/SEMA3 expression ratio and tumor microvascular density; consistent with the hypothesis that the angiogenesis in schwannomas was driven by activation of the VEGF pathway. In contrast, in this study, we found a robust expression of SEMA3 in meningiomas (NF2 associated and sporadic) and furthermore; there was no correlation between expression of VEGF pathway components and tumor microvascular density. These findings suggest that the VEGF pathway may not be an important driving force of angiogenesis in meningiomas and may explain the different response rates observed for the two types of tumors. Currently, prospective studies of bevacizumab (NCT01125046) and combination of bevacizumab and everolimus (NCT00972335) are underway for patients with sporadic recurrent or progressive meningiomas.

## Conclusion

NF2-related meningiomas were not effectively treated by bevacizumab in this analysis of NF2 patients treated for their progressive vestibular schwannomas. We did not identify any predictors of tumor response to bevacizumab in our cohort. Pathologic analysis of components of the VEGF angiogenesis pathway and of tumor microvascular density in meningiomas showed robust expression of SEMA3 (an angiogenesis inhibitor that was not expressed in schwannomas) and in contrast of our findings in schwannomas, there was no correlation between expression of VEGF pathway components and tumor microvascular density in meningiomas. These findings suggest that the VEGF pathway may not be a driving force in angiogenesis in meningiomas and may explain limited clinical response in meningiomas.

## Supporting Information

Text S1 **Methods for determining tumor volume and performing regression analysis.**
(DOC)
