# Supplementary material for: Bevacizumab Treatment for Meningiomas in NF2: A Retrospective Analysis of 15 Patients
Source: PLoS One. 2013 Mar 21;8(3):e59941. doi: 10.1371/journal.pone.0059941 (PMC3605344; doi:10.1371/journal.pone.0059941)
Supplement: Text S1 — Methods for determining tumor volume and performing regression analysis. (DOC) [file pone.0059941.s001.doc]

**Text S1**

*Methods*

When colliding tumors occurred between a meningioma and schwannoma, tumor limits were determined based on contrast and signal differences between the two tumors and best estimate of tumor borders. For tumors near venous and arterial structures the best estimate of tumor limit was also performed based on differences between flow void and tumor on the non-contrast images and differences between arterial and venous and tumor enhancement on the contrast-enhanced images. In several patients we were able to identify meningeal thickening, defined as an abnormal (enlarged) appearance of the meninges with no associated nodule or tumor. Meningeal thickening was not included in the analysis. Where tumor contour limit was adjacent to an area of meningeal thickening (dural tail), the tumor contour was tapered by best visual estimate of tumor limit without including the dural tail. In a single tumor in which an adjacent tumor cyst was present, the cyst volume was excluded from the analysis. When tumors had an intra-osseous component, intra-osseous extension of tumors was included up to the brain parenchyma and the limit of the subcutaneous tissues. If CT scans were available for analysis, these were used to help guide the limit of intra-osseous tumor extension. In a single case, a meningioma whose extraosseous component appeared greater than 50% mineralized, the tumor was excluded from the analysis. No other meningioma was excluded based on mineralization.

For tumors involving the skull base, determination of the correct tumor diagnosis based on radiographic appearance on MRI may be difficult. Tumors involving the orbit were analyzed for presence of meningeal attachment and were often considered to be meningiomas, as historically, when surgically removed, tumors involving these regions in NF2 patients are much more likely to represent meningiomas than cranial nerve schwannomas. When multiple skull base schwannomas were present and in close contact with the vestibular schwannomas (i.e. schwannomas involving cranial nerves III, V, VII, and IX), contrast enhancement of tumors was used to determine vestibular schwannoma limits. In one instance of a composite schwannoma that extended from CNs III-IX, the contours of the IAC (CNs VII and VIII) component of the schwannoma were determined by the upper and lower boundaries of the internal auditory canal, on all time points.

Although internal auditory canal protocols were available for all of the patients, vestibular schwannoma measurements were performed using T1-weighted post-contrast axial scans from whole brain MRI scans only, for consistency in the analysis of the correlation between vestibular schwannoma and meningioma response. Intra-cochlear extension of vestibular schwannomas was included in volumetric analysis when visualized.

The parameters of the MR images used in this analysis were as follows. For the T1 weighted whole brain images, both pre and post contrast, 1.5T, TR 700, TE 14, 320-256 (frequency encoding) x 224-192 (phase encoding) matrix, 3-4 slice thickness/skip 1 mm, 22-24 cm FOV, 1-2 NEX, and varied only slightly from patient to patient and between time points. The dose of gadolinium was 0.2 mL/kg. The parameters for the T2, FLAIR, susceptibility, DWI, and FIESTA sequences were as per standard departmental protocol.

Regression Model

Slope estimates from simple linear regression models with volume/response rate as the outcome and time as the predictor were used as the dependent (post treatment) and independent (pre treatment) variables and models on per tumor and per patients basis using linear regression models and linear mixed models with patients as random coefficient, respectively.
